# Unilateral-dominant reduction in muscle volume in female knee osteoarthritis patients: computed tomography-based analysis of bilateral sides

**DOI:** 10.1186/s13018-020-02074-x

**Published:** 2020-11-19

**Authors:** Ayumi Tsukada, Kentaro Uchida, Jun Aikawa, Shotaro Takano, Dai Iwase, Manabu Mukai, Masayuki Miyagi, Yuta Nanri, Gen Inoue, Masashi Takaso

**Affiliations:** 1 Department of Orthopedic Surgery, Kitasato University School of Medicine, 1-15-1 Minami-ku Kitasato, Sagamihara City, Kanagawa 252-0374 Japan; 2 Shonan University of Medical Sciences Research Institute, Nishikubo 500, Chigasaki City, Kanagawa 253-0083 Japan; 3 Department of Rehabilitation, Kitasato University Hospital, 1-15-1 Kitasato, Minami-ku, Sagamihara, Kanagawa 252-0375 Japan

**Keywords:** Computed tomography, Bilateral, Muscle volume, Muscle strength, Osteoarthritis

## Abstract

**Background:**

Muscle weakness is associated with osteoarthritis pathology. A recent study demonstrated that measuring muscle volume using computed tomography (CT)-based analysis and comparing bilateral muscles in the same patient allowed for accurate evaluation of muscle volume in unilateral hip osteoarthritis (OA) patients. Here, we evaluated muscle volume using CT-based analysis and compared bilateral muscles in knee OA (KOA) patients.

**Methods:**

CT images were obtained from 35 female radiographic KOA patients the day prior to total knee replacement surgery. Muscle volume (MV) was semi-automatically analyzed. Knee extension muscle strength (MS) was determined using a hand-held dynamometer. The severity of KOA patients’ clinical symptoms was examined using four domains of the Japanese Orthopedic Association (JOA) score. We compared the difference in MS (ΔMS) and MV (ΔMV) between the operated side (OS), which exhibited severe radiographic OA or severe pain, and the contralateral side (CS).

**Results:**

JOA score was significantly lower in the OS than CS. MV and MS were also significantly lower in the OS than CS. There was no correlation between MV and MS or between MV and MS as a percentage of body weight on either side. However, ΔMV was positively correlated with ΔMS and pain on walking in the JOA.

**Conclusions:**

We evaluated MV and MS using bilateral CT images of the legs of KOA patients. A reduction in MV was observed on the OS, and was correlated with a reduction in MS and pain on walking. Bilateral CT image analysis may be useful for evaluating the relationship between OA pathology and muscle atrophy.

## Background

Knee osteoarthritis (KOA) is a well-known musculoskeletal disorder and a key cause of disability, particularly in elderly individuals [1]. A number of biomechanical pathways likely play major roles in KOA. For example, excessive mechanical stress arising from either a reduction in load-bearing area on the surface of a joint or applying a heavy load can halt the repair of damaged joint tissue [2]. Muscle weakness is a key measure of the extent of disability in OA patients [3]. A number of reports have demonstrated that a reduction in lean mass in the lower limbs is common in OA patients [4], and this reduction is correlated with a heightened risk of falls [5–7]. Therefore, accurate evaluation of the muscles of KOA patients is important to better understand the relationship between the muscle and OA pathology.

Previous studies have proposed that translational studies that fill the gap between basic and clinical research using three-dimensional imaging data provide a basis for creating anatomical models of the human anatomy and may allow orthopedic surgeons to determine ideal practices before orthopedic surgery [8–10]. Computed tomography (CT) is used for various applications, including the evaluation of orthopedic tumors [11] and fractures [12], and preoperative planning of orthopedic surgery [13]. CT is also regularly used to quantify muscle volume (MV) at regions around the knee and hip [14–16]. In particular, the cross-sectional area (CSA) of the affected region determined from CT images is measured to evaluate MV [14–16]. However, these measurements vary widely and are dependent on the location at which the section is taken. In addition, muscle mass varies greatly from person to person due to differences in height or body mass index (BMI), making it difficult to conduct comparisons and accurately elucidate the effect of KOA on muscle mass. A recent study showed that muscle volume in unilateral hip OA patients could be accurately evaluated by measuring muscle volume using CT-based analysis and comparing bilateral muscles in the same patient [17].

Here, we evaluated muscle volume using CT-based analysis and conducted comparisons between bilateral muscles in KOA patients.

## Methods

The study protocol received ethics approval from the Institutional Review Board (IRB) for Clinical Research and Treatment of Kitasato University (IRB approval number: B20-133).

According to power analysis conducted with *α* = 0.05 and power = 0.80 using G*POWER3, 34, and 17 samples were needed to obtain a statistically significant difference in muscle strength (MS) and MV, respectively, between the operated and contralateral sides. Therefore, we obtained CT images from 35 female patients with radiographic KOA the day prior to total knee replacement surgery. Four domains of the Japanese Orthopedic Association (JOA) score, namely, pain on walking (domain I), pain on ascending or descending stairs (domain II), range of motion (domain III), and joint effusion (domain IV) [18], were used to evaluate symptom severity (Table 1).

**Table 1:** The Japanese Orthopedic Association (JOA) score for patients with knee osteoarthritis

**I. Pain on walking (total 30 points)**	
Walking 1 km or more usually with no pain, without regard for mild pain, or rarely feeling pain with certain activities	30
Walking 1 km or more regardless of pain	25
Walking 500 m or more, but less than 1 km without regard for pain	20
Walking 100 m or more, but less than 500 m without regard for pain	15
Walking indoors or nearby, but less than 100 m without regard for pain	10
Inability to walk	5
Inability to stand	0
**II. Pain on ascending or descending stairs (total 25 points)**	
No pain	25
Pain with handrails, but no pain with step-by-step ambulation	20
Pain relieved by using handrails	15
Pain with step-by-step ambulation, pain relieved by using handrails	10
Pain even with step-by-step ambulation and handrail use	5
Inability to ascend or descend because of pain	0
**III. Range of motion (total 35 points)**	
Squatting	35
Sideways or cross-legged sitting	30
Flexion or arc of motion of 110° or more	25
Flexion or arc of motion of 75° or more	20
Flexion or arc of motion of 35° or more	10
Flexion or arc of motion less than 35° including ankylosis or severe flexion contracture	0
**IV. Joint effusion (total 10 points)**	
No edema, no swelling	10
Occasional puncture required	5
Frequent puncture required	0

This table was translated by the authors with permission from the Japanese Orthopedic Association

### Muscle strength

Knee extension MS was determined using a hand-held dynamometer (μTas F-1; Anima, Tokyo) as participants were seated on a chair with their hips and knees flexed at 90°. Knee extension MS was expressed as the raw value in Newtons (N) and as a percentage of body weight (% BW). The difference in MS (ΔMS) between the operated side (OS), which exhibited severe radiographic OA or severe pain, and the contralateral side (CS) was calculated using the following equation:

$$ \mathrm{Difference}\ \mathrm{in}\ \mathrm{muscle}\ \mathrm{strength}\left(\Delta \mathrm{MS},\%\right)=\left({\mathrm{MS}}_{\mathrm{OS}}/{\mathrm{MS}}_{\mathrm{CS}}-1\right)\times 100 $$

### CT-based analysis of femoral muscle volume

**Fig. 1:**
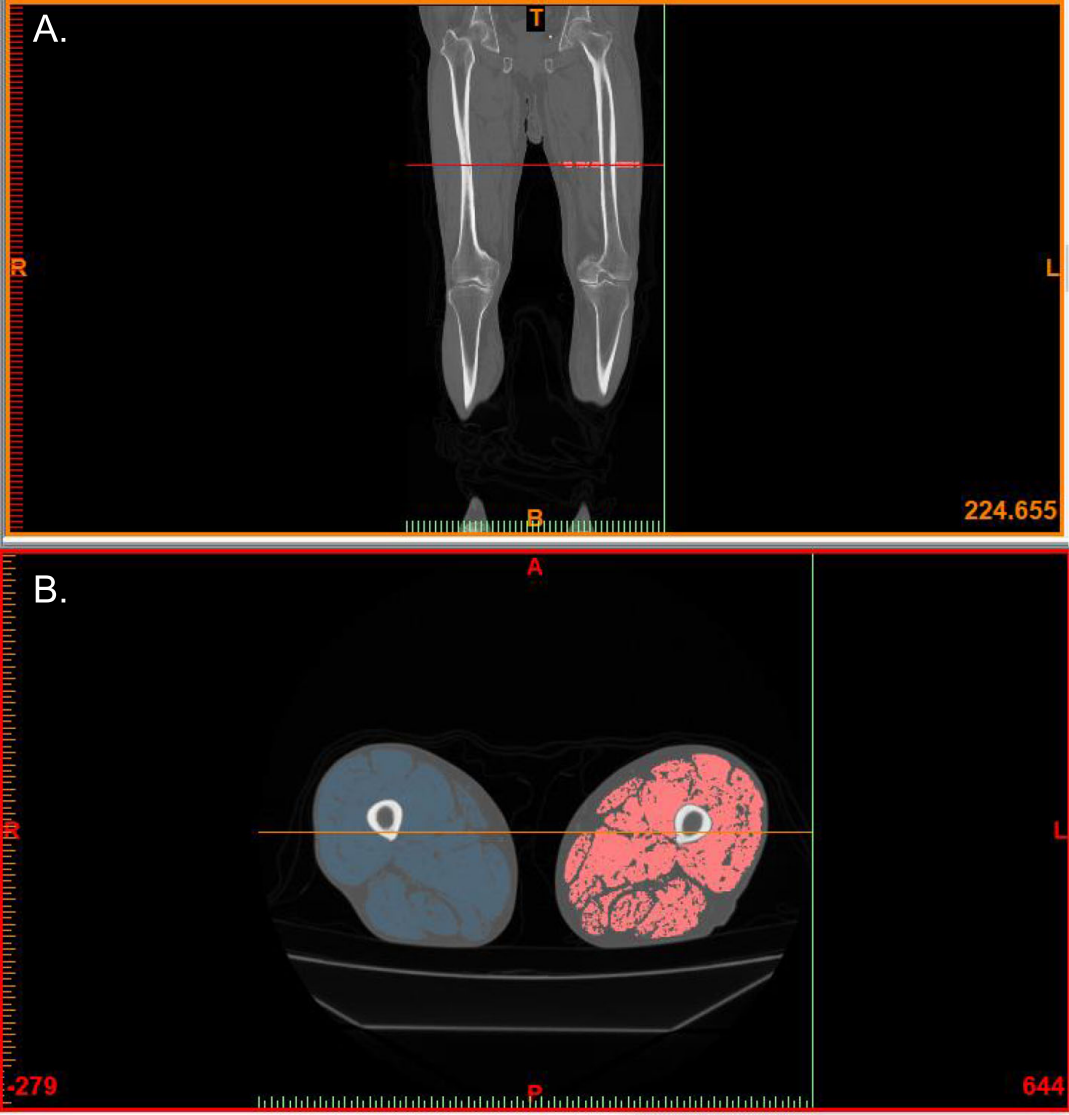
Estimation of muscle volume using CT-image analysis. **a** CT image of both legs of a patient. Red line indicates the location of a digital slice taken approximately 200 mm above the knee joint. **b** CT images of a cross section of bilateral muscles were semi-automatically analyzed in a 10-mm region of interest (ROI) using the MIMICS® software

Axial CT images were used to measure patients’ femoral MV. CT images comprising a 10-mm region of interest were taken 200 mm above the knee joint at 1-mm thickness and semi-automatically analyzed using the MIMICS® software (Materialise Japan Co., Ltd., Yokohama, Japan) (Fig. 1). Femoral MV was compared between OS and CS. Differences in MV between OS and CS were calculated using the following equation:

$$ \mathrm{Difference}\ \mathrm{in}\ \mathrm{muscle}\ \mathrm{volume}\ \left(\Delta \mathrm{MV},\%\right)=\left({\mathrm{MV}}_{\mathrm{OS}}/{\mathrm{MV}}_{\mathrm{CS}}-1\right)\times 100 $$

### Statistical analysis

Differences between OS and CS were examined using paired *t* test. The relationship between MV and MS was evaluated using Spearman’s correlation coefficient. A *P* value of < 0.05 was considered statistically significant. Statistical analysis was performed using the SPPSS software (Version 25.0; SPSS, IBM, Armonk, NY, USA).

## Results

### Study participant’s demographic information

The participants’ demographic and clinical information are provided in Table 2. Participants’ mean age was 72.4 ± 7.4 years and body mass index was 26.1 ± 4.1 kg/m^2^. There was no difference in the ratio of Kellgren/Lawrence (K/L) grades between the OS and CS. Total JOA score and the score for all four domains (pain on walking, pain on ascending or descending stairs, range of motion, and joint effusion) were significantly lower in the OS than CS.

**Table 2:** Patient demographic data

Parameter	Operated side	Contralateral side	*P* value
Age (years)	72.4 ± 7.4		
Height (cm)	149.0 ± 6.0		
Weight (kg)	57.9 ± 10.4		
BMI (kg/m^2^)	26.1 ± 4.1		
K/L grade (1, 2, 3, 4), *N*	0, 0, 6, 29	2, 4, 15, 14	*P* = 0.127
JOA score			
Total	54.6 ± 10.6*	73.4 ± 16.3	*P* < 0.001
Pain on walking	16.3 ± 5.9*	21.6 ± 7.3	*P* < 0.001
Pain on ascending or descending stairs	6.0 ± 4.8*	14.4 ± 7.9	*P* < 0.001
ROM	25.7 ± 3.4*	27.9 ± 0.7	*P* = 0.001
Joint effusion	6.6 ± 3.8*	9.6 ± 1.4	*P* < 0.001

Data indicate mean ± standard deviation unless otherwise indicated

*BMI* body mass index, *K/L* Kellgren/Lawrence grade, *JOA* Japanese Orthopedic Association, *ROM* range of motion

*P < 0.05 versus contralateral side

### Muscle strength and volume

**Fig. 2:**
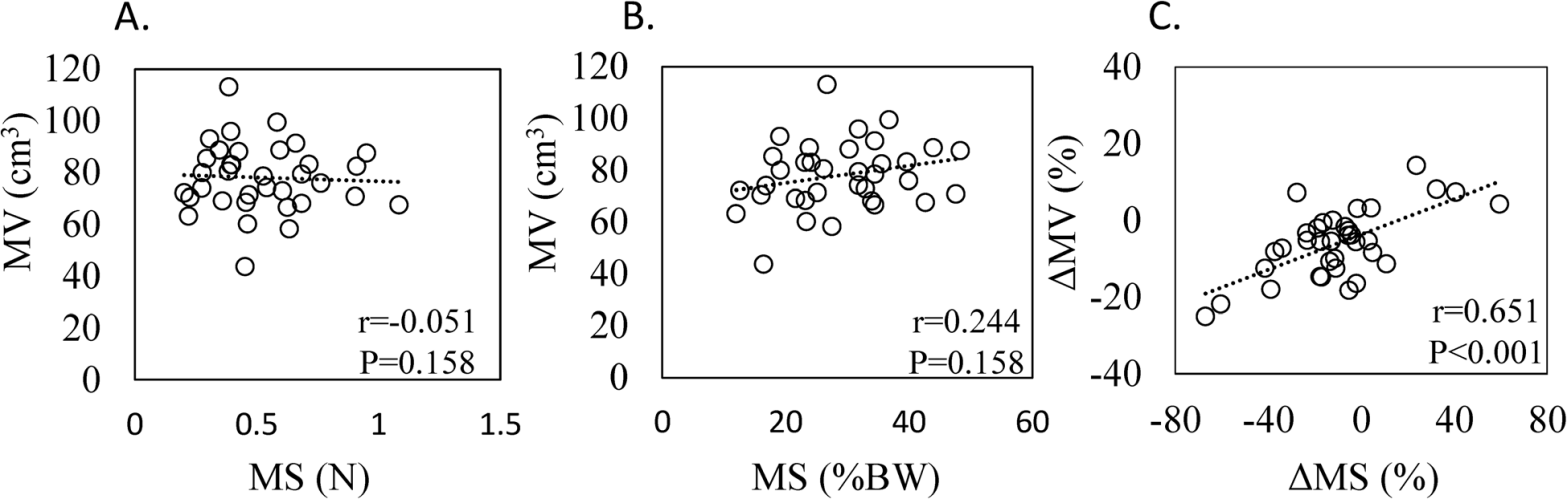
Relationship between muscle volume and muscle strength. Graphs show the correlation between **a** muscle volume (MV) and muscle strength (MS), **b** MV and MS as a percentage of body weight (%BW), and **c** the difference in MV (ΔMV) and MS (ΔMS)

MS and MS as a percentage of body weight were significantly reduced in the OS compared to CS (*P* = 0.001; Table 3). MV was also significantly reduced in the OS compared to CS (*P* = 0.001; Table 3). No correlation was found between MV and MS (Fig. 2a) or MV and MS as a percentage of body weight on the OS (Fig. 2b). However, ΔMV was positively correlated with ΔMS on the OS (*r* = 0.651, *P* < 0.001; Fig. 2c).

**Table 3:** Muscle strength and muscle volume

	Operated side	Contralateral side	Difference (%)	*P* value
Muscle strength (*N*)	0.52 ± 0.22*	0.61 ± 0.25	−10.14 ± 25.18	*P* = 0.001
% Muscle strength to body weight (% BW)	28.78 ± 9.71*	33.62 ± 11.57		*P* = 0.001
Muscle volume (cm^3^)	77.95 ± 13.11*	83.03 ± 12.09	−6.0 ± 8.9	*P* = 0.001

Data indicate mean ± standard deviation unless otherwise indicated

*P < 0.05 versus contralateral side

### Relationship between clinical score, muscle strength, and muscle volume

**Fig. 3:**
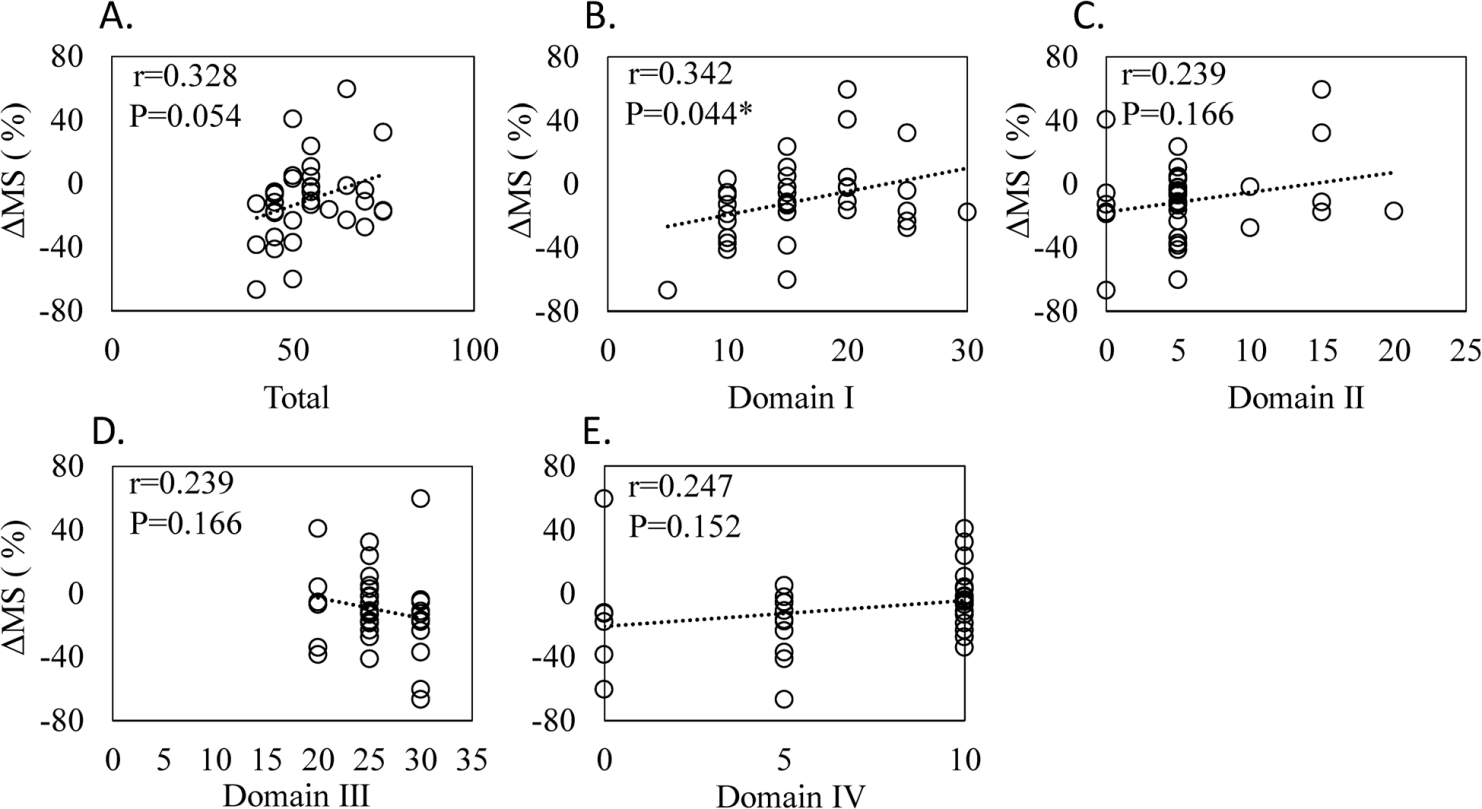
Correlation between a reduction in unilateral muscle strength and the Japanese Orthopedic Association (JOA) score. Correlation between a reduction in unilateral muscle strength (ΔMS) and **a** total JOA score (total), **b** domain I (pain on walking), **c** domain II (pain on ascending or descending stairs), **d** domain III (range of motion), and **e** domain IV (joint effusion)

**Fig. 4:**
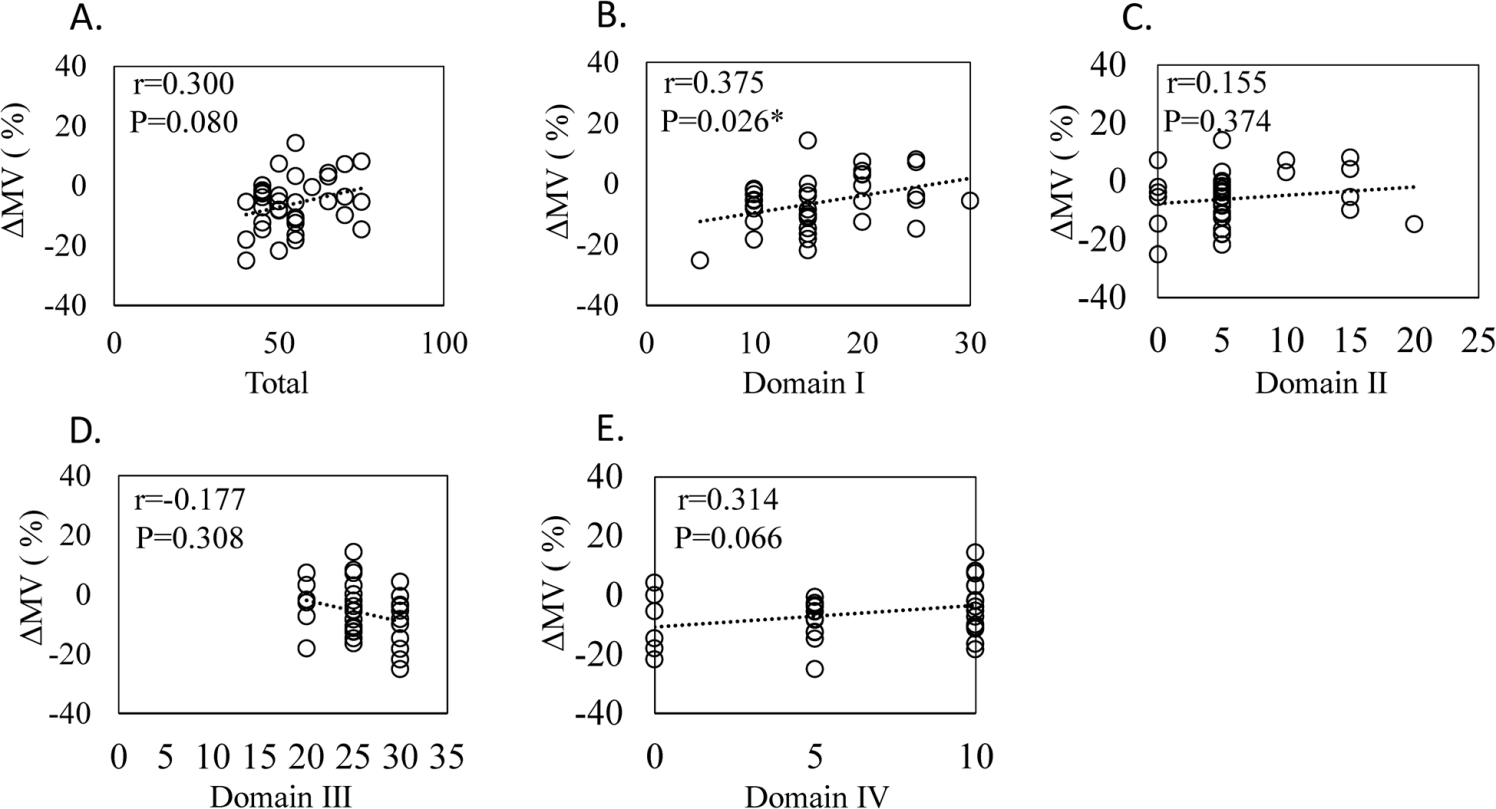
Correlation between reduction in unilateral muscle volume and Japanese Orthopedic Association (JOA) score. Correlation between a reduction in unilateral muscle volume (ΔMV) and **a** total JOA score (total), **b** domain I (pain on walking), **c** domain II (pain on ascending or descending stairs), **d** domain III (range of motion), and **e** domain IV (joint effusion)

There was no correlation between the total JOA score and the score for three domains (pain on ascending or descending stairs, range of motion, and joint effusion) and ΔMS (Fig. 3a, c–e) or ΔMV (Fig. 4a, c–e). In contrast, both ΔMS and ΔMV were significantly correlated with domain I (pain on walking) in JOA (ΔMS, *r* = 0.342, *P* = 0.044, Fig. 3b; ΔMV, *r* = 0.375, *P* = 0.026; Fig. 4b).

## Discussion

Previous studies have shown that KOA patients exhibit a reduction in muscle mass in the lower limbs compared to age-matched healthy controls [6, 19, 20]. However, a cohort study reported a weak association between bone mass, measured based on bioimpedance analysis (BIA) using a body composition analyzer, on muscle strength in KOA patients [19]. Consistent with previous reports, we found no correlation between MS and MV. However, bilateral MV analysis using CT images taken from KOA subjects showed a unilateral decrease in MV on the side with severe OA and that this reduction was strongly correlated with a reduction in MS. CT-based analysis of bilateral sides may therefore be a useful tool for evaluating muscle weakness in KOA patients due to the exclusion of parameters such as age and BMI, which affect muscle mass and strength (Table 4).

**Table 4:** Advantages and limitations of this study

Advantages	Limitations
• CT-based analysis of bilateral muscle volume accurately reflects muscle strength in patients with KOA. • CT-based analysis of bilateral muscle volume enables exclusion of parameters such as age and BMI, which affect muscle mass and strength.	• The analyzed muscle comprises several different muscles, including the rectus femoris, biceps femoris, and adductor longus. • This study was a cross-sectional study. • Patients with bilateral knee osteoarthritis were included among the participants.

*CT* computed tomography, *KOA* knee osteoarthritis, *BMI* body mass index

There is a logical biomechanical explanation for the association between leg muscle mass and knee symptoms. Periarticular muscles, which keep injured and degenerated knees structurally stable and supported, with greater mass relative to total body mass provide better stability, resulting in less pain. Several studies examining changes to the muscles in the lower limbs have shown that low impact exercises like swimming and cycling are beneficial for reducing pain in patients with KOA [21, 22]. Therefore, low impact therapeutic approaches may be important for relieving pain in KOA patients.

Several reports have suggested that unilateral mechanical unloading is a cause of muscle reduction [23–25]. Studies in humans have reported that, as a result of unilateral lower limb unloading, there is a 7% decrease in muscle CSA after 21 days [24] and a 16% decrease after 35 days [23, 25]. In the present study, MV was reduced by approximately 6% on the side with severe OA, and this reduction was associated with pain on walking. Underuse of the muscle due to pain may therefore explain the unilateral reduction in MV in KOA patients.

This study has several limitations (Table 4). First, the analyzed muscle comprises several different muscles, including the rectus femoris, biceps femoris, and adductor longus. Assessment of individual muscles is necessary to obtain a practical understanding of functional disability in patients with KOA. Second, our study was a cross-sectional study. Longitudinal studies are needed to clarify the relationship between OA development and muscle reduction. Finally, patients with bilateral KOA were included among the participants in this study.

## Conclusion

We evaluated MV using bilateral CT images of the legs of patients with severe knee pain. MV was reduced on the side with severe OA and this reduction was correlated with a decrease in MS and pain. Bilateral CT image analysis may be useful for evaluating the link between OA and muscle pathology due to the ability to exclude parameters such as age and BMI, which affect muscle mass and strength. Further investigation using a longitudinal study may clarify the relationship between OA development and muscle reduction.

## Data Availability

The datasets supporting the conclusions of this article are included within the article. The raw data can be requested from the corresponding author.

## Abbreviations

BIA: Bioimpedance analysis
BMI: Bone mass index
CSA: Cross-sectional area
CS: Contralateral side
CT: Computed tomography
IRB: Institutional Review Board
JOA: Japanese Orthopedic Association
OA: Osteoarthritis
OS: Operated side
%BW: Percentage of body weight
MV: Muscle volume
MS: Muscle strength
